# Who are the key players? Listeners vs spreaders vs others

**DOI:** 10.1371/journal.pone.0286369

**Published:** 2023-05-31

**Authors:** Sumin Kim, Kyu-Min Lee, Euncheol Shin

**Affiliations:** KAIST College of Business, Seoul, Republic of Korea; Chongqing Three Gorges University, CHINA

## Abstract

The literature on social learning examines how people learn from their neighbors and reach a consensus. The DeGroot social learning model describes the social learning process as one in which people form their opinions by taking a weighted average of their neighbors’ opinions. In the model, the influence structure is represented by a single matrix. In this paper, we empirically identify the role of the hub and authority centralities based on this matrix using data on microfinance adoption in rural Indian villages. Controlling for other well-known centrality measures, authority centrality is positively associated with final adoption rates in the villages, but hub centrality is not. Furthermore, we find that authority centrality is the most informative variable predicting microfinance diffusion success from LASSO regressions.

## Introduction

In a variety of contexts, prior to an actual choice decision, people often gather information from their acquaintances, such as friends, family, coworkers, and neighbors [1]. For example, voters exchange their opinions about political candidates before casting their ballots [2]. In addition, before subscribing to a particular mobile carrier, consumers may collect information regarding the quality of multiple carriers [3]. Moreover, when it comes to developing countries, citizens collect information about microfinance provided by banks and international organizations [4]. As such, political candidates, carrier firms, and organizations might want to know how people influence and are influenced by one another to target a specific group of people to maximize their objectives, such as vote count, subscription rates, or program adoption. For this targeting intervention, the central question is how to identify key players in the communication network [5, 6].

Identifying key players in a network is not only a theoretical question but also an empirical one that depends on a particular context [7]. In addition, it relies on whether agents strategically interact without a third party’s intervention or if there is an intervention designer that has information about the interaction structure represented by a social network. In the literature on social and economic networks, there have been a number of substantial centrality measures (e.g., degree, eigenvector, and Bonacich centralities) that explain the success and failures of diffusions in social networks by empirical studies [1, 4].

In this paper, we focus on the *hub* and *authority* centralities developed by Kleinberg [8] that have been less explored in the social learning context. As a social learning model, we consider that of DeGroot, in which agents form their opinions as a weighted sum of their neighbors’ opinions [9–11]. The agents’ opinion exchanges in the model are summarized by a single matrix called the *influence matrix*. The hub and authority centralities are then defined as the left and right singular vectors associated with the largest singular value of the influence matrix [8]. In other words, the hub centralities capture the influence of the agents on other agents’ opinion formation, and the authority centralities capture the connectivity of the agents to those with strong influence.

A recent paper by Jeong and Shin showed that these centralities are relevant to an optimal target intervention problem in a general framework [6]. However, in the current paper, we empirically test whether these two centrality measures explain the success of the microfinance adoption behavior in [4], in which the authors considered the diffusion of microfinance in rural villages in India. Before the introduction of their microfinance program, they investigated the household-level social networks and their adoption behavior in 43 villages. From their dataset, for each village, we can construct an undirected social network of households where each link between two households represents a social connection like helping each other with agricultural work or lending or borrowing money. We then construct an influence matrix of the agents to identify the hub and authority centralities of the agents who obtain the microfinance information at the outset. Those agents are called *leaders*, and we test which of the leaders’ two centrality measures are related to the final adoption rate of microfinance in the villages.

We find that authority centrality plays a central role in explaining the success of diffusion in the data, but hub centrality does not. This observation remains even after controlling for other variables studied in the original paper. Furthermore, by employing the least absolute shrinkage and selection operator (LASSO) regressions, we show that authority centrality is the most critical variable explaining the success of the adoption rate in a village.

Our results provide a notable practical implication. Note that the calculation of the authority centrality does not require detailed adoption data, which was necessary for calculating communication centrality in [4]. Consequently, from simple social network data, one can identify the authority centralities before starting the spread of the agents’ behavior in a social network. Calculating authority centrality is equivalent to calculating singular vectors associated with the largest singular value, and several algorithms can efficiently calculate it [12, 13]. Therefore, this paper specifies a new direction for network research by emphasizing the hub and authority centrality, especially the authority centrality among various network characteristics.

## Materials and methods

We begin by presenting the theoretical background of the network centrality measure used in the latter empirical analysis. Throughout the paper, we refer to a book by Jackson [1] for formal definitions of the terminologies other than the definitions of hub and authority centralities. We then introduce the data and empirical strategy for empirical analysis.

### Theoretical background

#### Hub and authority centralities

**Fig 1:**
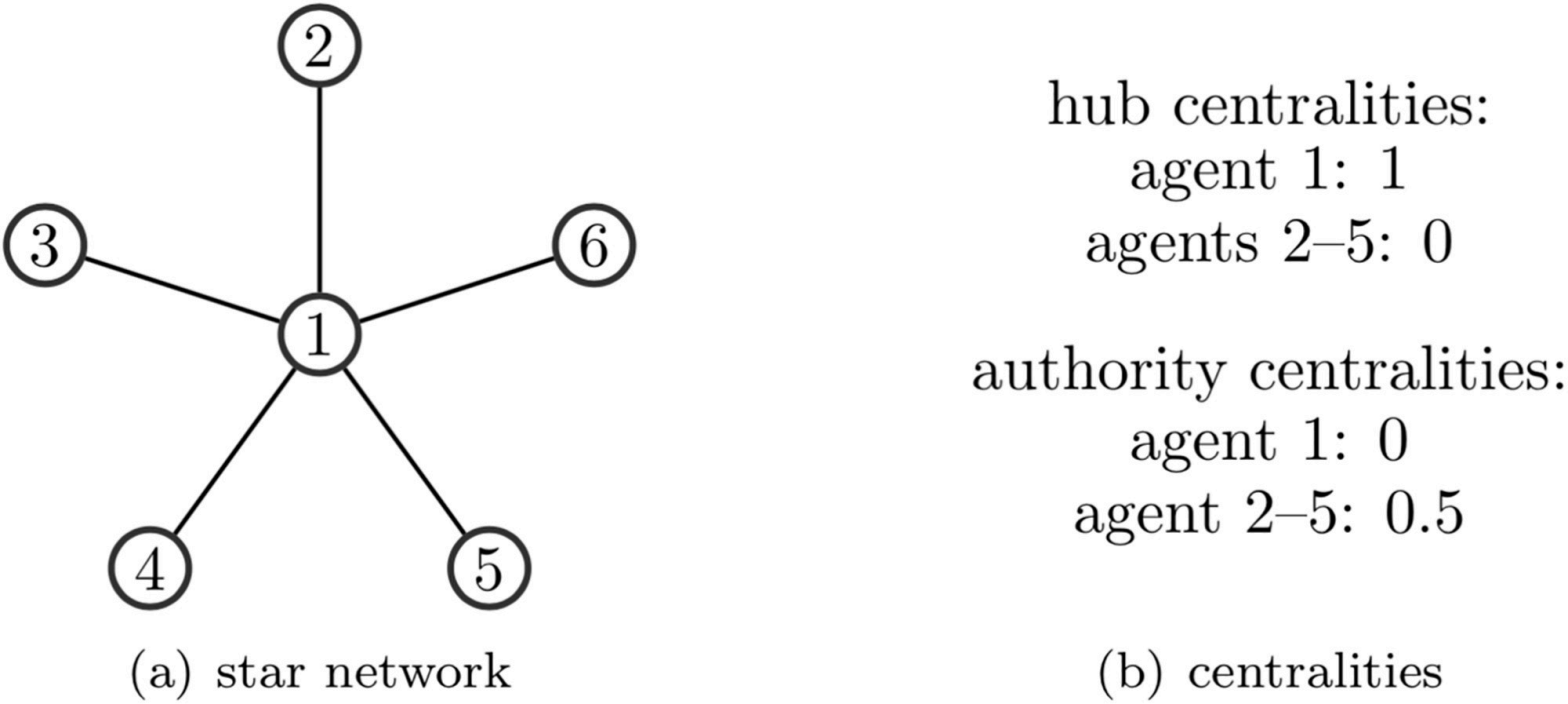
Example of five agents. In (a), two agents are connected by a link if they are mutual friends. Thus, the network is undirected. In (b), we highlight the authority and hub centralities of agent 1 and the other agents. Agent 1 has a distinguished hub centrality as everyone listens to him. On the contrary, as he is not linked to any agent with nonzero hub centrality, his authority centrality is zero.

Hub centrality has a character of large out-degree; therefore, it is the measure of how it links to another neighbor as a spreader. In contrast, authority centrality has a character of in-degree, representing how well it is linked to other neighbors’ opinions as an informed listener. Kleinberg’s research [8] described hub nodes as the nodes that can link to good authority nodes and authority nodes as the nodes which can be linked to good hub nodes. Fig 1 illustrates an example of a undirected star network’s hub and authority centrality measures.

From social network data consisting of *n* agents, the hub and authority centralities are defined as follows. An undirected social network of *n* agents is represented by an *n* × *n* symmetric (0, 1)-matrix *A*, where its (*i*, *j*) entry represents that agent *i* and agent *j* are connected by a link. If they are connected by a link, the entry becomes one; otherwise, the entry is zero. This matrix is called the adjacency matrix of the network. The degree matrix of *A* is denoted by *D*, which is an *n* × *n* diagonal matrix. Its *i*th diagonal entry represents the degree of agent *i* counting the number of non-zero entries in the *i*th row of the adjacency matrix *A*. We assume that every agent of the network has at least one neighbor. Thus, the degree matrix is invertible.

For a given social network represented by its adjacency matrix *A*, the influence matrix *T* is defined as *T* = *D*^−1^*A*. For instance, the adjacency and influence matrices for the social network in Fig 1(a) are

$$\begin{array}{c} A=(\begin{array}{ccccc} 0 & 1 & 1 & 1 & 1\\ 1 & 0 & 0 & 0 & 0\\ 1 & 0 & 0 & 0 & 0\\ 1 & 0 & 0 & 0 & 0\\ 1 & 0 & 0 & 0 & 0 \end{array})\;\text{and}\;T=(\begin{array}{ccccc} 0 & 0.25 & 0.25 & 0.25 & 0.25\\ 1 & 0 & 0 & 0 & 0\\ 1 & 0 & 0 & 0 & 0\\ 1 & 0 & 0 & 0 & 0\\ 1 & 0 & 0 & 0 & 0 \end{array}). \end{array}$$ (1)

DeGroot’s model assumes that agents update their beliefs according to a simple heuristic learning rule. Specifically, let *b*^0^ be the initial opinion vector of the agents. Then, the agents’ updated opinion vector *b* is expressed by *b* = *Tb*^0^. This model delivers a fairly complete characterization of learning dynamics as a function of network structure, as shown in a series of theoretical papers [10, 14]. Moreover, recent lab experimental works have supported this choice of social learning model [15–17].

Hub and authority centralities are then defined through a singular value decomposition of the influence matrix *T*. By applying the singular value decomposition, we can find that there are matrices *U*, *S*, *V* such that *T* = *USV*′, where *V*′ is the transpose of matrix *V*. For *U*, *S*, and *V*, we have the following properties:

1. *S* = *diag*(*s*_1_, …, *s*_*n*_), where *s*_1_ ≥ *s*_2_ ≥ ⋯ ≥ *s*_*n*_ ≥ 0 and *s*_*k*_ ≥ *s*_*k*+1_ for all *k*;
2. *U* and *V* are normal matrices;
3. the *k*th column vector of *U* is a left singular vector of *T*: *Tv*^*k*^ = *s*_*k*_*u*^*k*^;
4. the *k*th column vector of *V* is a right singular vector of *T*: (*u*^*k*^)′*T* = *s*_*k*_(*v*^*k*^)′.

The first column of the right matrix *V* is said to be the hub centrality, and the first column of the left matrix *U* is referred to as the authority centrality, following [8]. As the singular value decomposition exists for any well-defined influence matrix *T*, the hub and authority centralities are also well-defined [12]. We refer to Jeong and Shin’s research paper for more details on the singular value decomposition and the centrality measures in the social learning context with examples [6].

#### Other network centralities

To evaluate the explanatory power of the hub and authority centralities, we consider the following series of well-documented centrality measures in the literature on social and economic networks:

- degree centrality: the degree centrality of an agent counts the number of neighboring agents linked to agent *i*.
- betweenness centrality: the betweenness centrality of an agent is the number of the shortest paths between other agents that pass through the agent.
- Bonacich centrality: Bonacich centrality of an agent is based on the number of walks emanating from the agent [18–20]. It requires a discount factor parameter. As in the research by Banerjee et al. [4], we use the parameter of 0.8 × λ_1_(*A*), where λ_1_(*A*) is the largest eigenvalue of adjacency matrix *A*.
- decay centrality: The decay centrality of an agent measures the distance that takes into account the decay in traveling along shortest paths in the network [1]. We use the decay centrality with parameter *p* = 0.18 as in Banerjee et al. [4].
- closeness centrality: the closeness centrality of an agent is the average length of the shortest path between the agent and all other nodes in the network.
- diffusion centrality: Diffusion centrality of an agent is based on a specific dynamic diffusion process starting at the agent. Depending on a choice of a parameter, it can be proportional to the degree centrality or the Bonacich centrality.
- communication centrality: An agent’s diffusion centrality calculates the number of times that all agents taken together will be aware of the opportunity to participate in microfinance [4]. Calculation of the communication centrality also requires a specific dynamic diffusion process.
- eigenvector centrality: the eigenvector centrality of an agent measures their wide-reaching influence within a given network, taking into account the importance of their connections with other influential agents.

We refer to Banerjee et al. [4], Bloch et al. [21], and Jackson [1] for the formal definitions of these centrality measures. Among the above seven listed centrality measures, the communication centrality developed by Banerjee et al. [4] is different from the other centrality measures of (i)–(vi) and (viii) in that communication centrality requires the behavioral data of the agents in a social network. In other words, except for the communication centrality, the amount of information to calculate the centralities of agents is that of the social network data alone.

### Data

We use the dataset collected by Banerjee et al. [4], which has been used for several empirical studies in the literature [22–26]. The original dataset is publicly available at https://web.stanford.edu/~jacksonm/Data.html; alternatively, to replicate the results in this paper, one can use the dataset we include in the Supporting Information of the current paper, which is also available on the corresponding author’s website (https://sites.google.com/site/euncheolshin00). The original dataset includes a comprehensive social network of households in rural Indian villages. It also contains the households’ participation rates when the microfinance program was introduced. Specifically, the microfinance institution collected the network data of 43 villages before introducing its services. Based on the data, the microfinance institution chose leaders as an injection point and relied on the word-of-mouth communication of the leaders and other participants. After receiving the information on final participation, the researchers developed the communication centrality of the households, including the leaders who had initially been chosen. Since each village is isolated from the other villages, each household’s centrality is calculated from the social network containing it. Banerjee et al. showed that the communication centrality of the leaders was positively associated with the participation rates in the villages, even after controlling for other centralities and characteristics of the leaders [4].

We find in the data that there is no isolated agent, and so the assumption to construct the influence matrix *T* is satisfied because *D*(*A*) is invertible. As for normalization, after calculating the average of the centralities of the leaders in each social network, we divide every leader’s centrality by the average. Table 1 gathers summary statistics of the data.

**Table 1:** Summary statistics.

Datasets:	Mean (1)	Std. (2)	Min (3)	Max (4)
**Panel A: Network Characteristics**				
Hub centrality	0.006	0.003	0.002	0.021
Authority centrality	0.004	0.002	0.001	0.008
Degree centrality	18.101	3.784	11.889	28.053
Betweenness centrality	0.030	0.009	0.016	0.069
Bonacich centrality	4.341	0.419	3.311	5.191
Decay centrality	5.413	1.085	3.544	8.097
Diffusion centrality	5.485	1.745	2.063	10.503
Closeness centrality	0.431	0.034	0.362	0.491
Communication centrality	0.065	0.045	0.002	0.185
Eigenvector centrality	0.074	0.017	0.044	0.123
**Panel B: Other Individual Characteristics**				
Participation rate	0.185	0.084	0.068	0.438
Fraction of leaders	0.121	0.031	0.064	0.193
Average savings	1.613	0.098	1.354	1.837
Average self-help group participation	0.207	0.084	0.014	0.354
Fraction of GM caste	2.508	0.373	1.068	3.030

Table notes: The above table reports descriptive statistics of the data for latter regression analysis. Column (1) shows the means, column (2) represents the standard deviations, column (3) indicates the minimum value, and column (4) denotes the maximum value. Panel A gathers network centralities, and panel B collects other individual-level characteristics of the leaders. The unit of observations is the average of each variable in a villages; as such, the number of observation for each variable is the same as 43.

**Fig 2:**
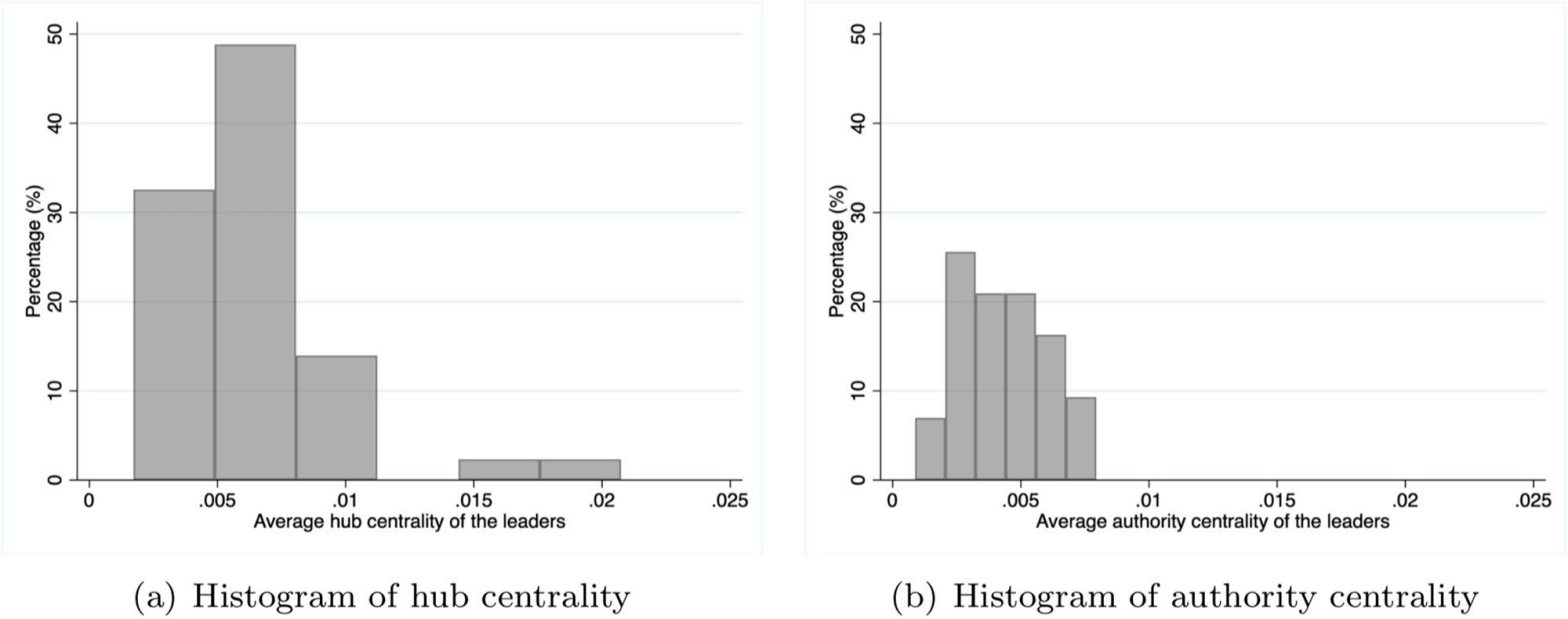
Histograms of hub and authority centralities. In (a), The horizontal axis indicates the leaders’ average hub centrality. The vertical axis represents the percentage of the average hub centrality of the leaders contained in each bin. In (b), The horizontal axis indicates the average of authority centrality of the leaders. The vertical axis represents the percentage of the average hub centrality of the leaders contained in each bin.

Panel A in Table 1 collects summary statistics of the network characteristics of the leaders in 43 social networks. For each network characteristic in a social network, the average centrality of leaders is calculated. The mean, standard deviation, minimum value, and maximum value are reported. The hub and authority centralities have similar statistics, and the histograms of the two centralities are shown in Fig 2. One can find that for both centralities, heterogeneity exists across the leaders in the 43 networks. The histogram of the hub centrality is more skewed than the distribution of authority centrality. The correlation coefficient of the two centralities is 0.174, which indicates that a village having a group of leaders of high hub centrality does not necessarily mean a high average of authority centrality, and vice versa. The summary statistics of the other centralities are the same as in [4].

Panel B in Table 1 gathers summary statistics of the other characteristics. There is variation in the participation rate among the social networks, and it is distributed between 6.8% and 43.8%. On average, about 12.1% of leaders were selected to spread the information to their neighbors through social networks.

### Empirical strategy

To examine the relationship between the participation rates of the microfinance program and the two centralities of the leaders, we consider the following two regression equations:

$$\begin{array}{cc} & y_{i}=\alpha +\beta_{1}Hub_{i}+\beta_{2}Communication_{i}+\beta_{3}Eigen_{i}+X_{i}^{\prime }\gamma +Z_{i}^{\prime }\delta +\varepsilon_{i}, \end{array}$$ (2)

$$\begin{array}{cc} & y_{i}=\alpha +\beta_{1}Authority_{i}+\beta_{2}Communication_{i}+\beta_{3}Eigen_{i}+X_{i}^{\prime }\gamma +Z_{i}^{\prime }\delta +\varepsilon_{i}, \end{array}$$ (3)

where for each social network *i*, *y*_*i*_ is the participation rate, *Hub*_*i*_ is the average hub centrality of the leaders, *Authority*_*i*_ is the average authority centrality of the leaders, *Communication*_*i*_ is the average communication centrality of the leaders, *Eigen*_*i*_ is the average eigenvector centrality of the leaders, *X*_*i*_ is a vector of other average centralities of the leaders, *Z*_*i*_ is the vector of the fraction of leaders, the average saving of the leaders, the average self-help group participation rate, and the fraction of GM caste. *ε*_*i*_ is an error term. The coefficient of interest is *β*_1_, which captures the changes in the participation rate in social network *i* in the change in the average centrality of the interest. Another coefficient of interest is *β*_2_, which captures the changes in the participation rate in social network *i* in the change in the average communication centrality, the crucial centrality suggested by [4]. For statistical inference, we calculate robust standard errors. We run regression models (1) and (2) by including control variables and vectors one by one.

We also conduct LASSO regression [27] to identify the substantial predictive power of the hub and authority centrality measures. A LASSO regression is similar to the regular MSE regression but includes the sum of the absolute values of each coefficient in the formula to also minimize the size of the coefficients (i.e., *L*^1^-norm penalty). If the sum of the absolute values of the coefficients is assumed to be zero, then every coefficient becomes zero. In contrast, if the sum of absolute values is assumed to be smaller than a very large number, then the constraint is not effective, and each coefficient of the variables becomes identical to the coefficient without constraint. Therefore, by examining how the coefficients change by relaxing the constraint, one can see the prediction power of the variables.

In our LASSO analysis, the main task is to identify the rank of explanatory variables predicting the participation rate in the data. Due to the small sample size of 43 in our dataset, we do not conduct a model selection task; instead, we would like to show that the average authority centralities of leaders have the largest prediction power by plotting the coefficient paths as a function of *L*^1^-norm of the standardized coefficient vector. As is shown in the following section, we run the regression only for the authority centrality because hub centrality is not associated with the participation rate with a statistical significance. As such, we consider the following constrained minimization problem:

$$\begin{array}{cc} \underset{\beta }{\text{min}}\; & \sum_{i}{\left(y_{i}-{\hat{y}}_{i}\right)}^{2} \end{array}$$ (4)

$$\begin{array}{cc} \text{subject}\;\text{to}\; & \|\beta \|\leq t, \end{array}$$ (5)

where ${\hat{y}}_{i}=\alpha +\beta_{1}Authority_{i}+\beta_{2}Communication_{i}+\beta_{3}Eigen_{i}+X_{i}^{\prime }\gamma +Z_{i}^{\prime }\delta +\varepsilon_{i}$ in (3) and *β* = (*α*, *β*_1_, *β*_2_, *β*_3_, *γ*′, *δ*′) in (4) is the vector collecting all the coefficients to predictor ${\hat{y}}_{i}$, and *t* ≥ 0 is a prespecified free parameter that determines the degree of *L*^1^ regularization. The explanatory variables are the same as in the previous specification for the regression analysis. We solve the above minimization problem by including explanatory variables in the expression of ${\hat{y}}_{i}$ one by one. For a given set of explanatory variables, we increase the value of *t* from zero in order to see how the coefficients of the explanatory variables vary.

## Results

### OLS analysis

Table 2 gathers results on the relationship between the hub centrality and the adoption rate with regression Eq (3). Column (1) is the baseline regression result that finds that there is no statistically significant relationship between the two variables. In column (2), it turns out that the communication centrality still explains successful adoptions in the villages after controlling for the hub centrality of the leaders. This result then remains the same after adding additional control variables of the network centralities and characteristics of the leaders. Column (5) includes the eigenvector centrality of the leaders. While there is no significant change in the magnitudes and standard errors of the estimates for the two previous centrality measures compared to column (4), the explanatory power of the communication centrality diminishes after the inclusion of the eigenvector centrality. It is worth noting that the full specification of column (5) includes 13 variables, which is a relatively high number compared to the sample size of 43. Thus, the loss of explanatory power of the communication centrality does not necessarily invalidate the findings in Banerjee et al. [4].

**Table 2:** Hub centrality and adoption rate.

Variables	(1)	(2)	(3)	(4)	(5)
Hub centrality	−0.592 (3.534)	−1.894 (2.253)	−2.329 (2.276)	−2.736 (3.078)	−3.000 (2.933)
Communication centrality		0.898*** (0.320)	0.657* (0.367)	0.752* (0.387)	0.695 (0.424)
Eigenvector centrality					3.521 (2.341)
Other network centralities	No	No	Yes	Yes	Yes
Other characteristics	No	No	No	Yes	Yes
Constant	0.188*** (0.023)	0.138*** (0.022)	−0.048 (0.132)	0.520* (0.290)	0.831** (0.333)
Observations	43	43	43	43	43
R-squared	0.001	0.232	0.443	0.510	0.537

Table notes: Robust standard errors in parentheses. Network characteristics include the average degree of leaders, the average between centrality of leaders, the average Bonacich centrality, the average decay centrality, and the average closeness centrality. Other characteristics include the fraction of nodes that are leaders, the average savings, the average self-help group participation rate, and the fraction of GM caste.

***p < 0.01,

**p < 0.05,

*p < 0.1

Table 3 shows that the authority centrality of the leaders is highly correlated with the success of microfinance adoption. Column (1) shows that the authority centrality is positively associated with the adoption rate. In magnitude, one standard deviation increase in the average authority centrality of the leaders means about a 4.7 percentage point increase in the final adoption rate in the data (*p*-value<0.001). The positive relationship remains the same even when the community centrality is additionally added as a control variable in column (2). The significant impact of the authority centrality remains in column (4) containing all the other variables of network centralities (except the eigenvector centrality) and characteristics of the leaders. In magnitude, one standard deviation increase in the average authority centrality of the leaders means about a 2.75 percentage point increase in the final adoption rate in the data (*p*-value<0.097), which is about one-third of the standard deviation of the participation rate variable. This magnitude is slightly greater than the one for the communication centrality as one standard deviation increase in the average communication centrality of the leaders implies about a 2.67 percentage point increase in the adoption rate in the data (*p*-value<0.084).

**Table 3:** Authority centrality and adoption rate.

Variables	(1)	(2)	(3)	(4)	(5)
Authority centrality	26.798*** (7.298)	20.721*** (6.470)	16.078* (8.735)	15.602* (9.121)	14.599 (9.152)
Communication centrality		0.500* (0.275)	0.515 (0.315)	0.590* (0.330)	0.554 (0.358)
Eigenvector centrality					2.992 (2.159)
Other network centralities	No	No	Yes	Yes	Yes
Other characteristics	No	No	No	Yes	Yes
Constant	0.070** (0.023)	0.064** (0.026)	0.003 (0.130)	0.560* (0.282)	0.821** (0.343)
Observations	43	43	43	43	43
R-squared	0.316	0.373	0.484	0.547	0.566

Table notes: Robust standard errors in parentheses. Network characteristics include the average degree of leaders, the average between centrality of leaders, the average Bonacich centrality, the average decay centrality, and the average closeness centrality. Other characteristics include the fraction of nodes that are leaders, the average savings, the average self-help group participation rate, and the fraction of GM caste.

***p < 0.01,

**p < 0.05,

*p < 0.1

Interestingly, the statistical significance of authority centrality does not fall behind the statistical significance of communication centrality. These results show that the authority centrality may have higher predictive power than the communication centrality to explain the success of microfinance adoption.

In column (5), we include the average eigenvector centrality of the leaders. Similar to the previous analysis with the hub centrality, there is no significant change in the magnitudes and standard errors of the estimates for the two previous centrality measures, compared to column (4). However, the authority centrality loses its explanatory power, which could be attributed to the limited sample size of the data. Therefore, we conduct a LASSO regression to compare the predictive power of the three primary centrality measures.

**Fig 3:**
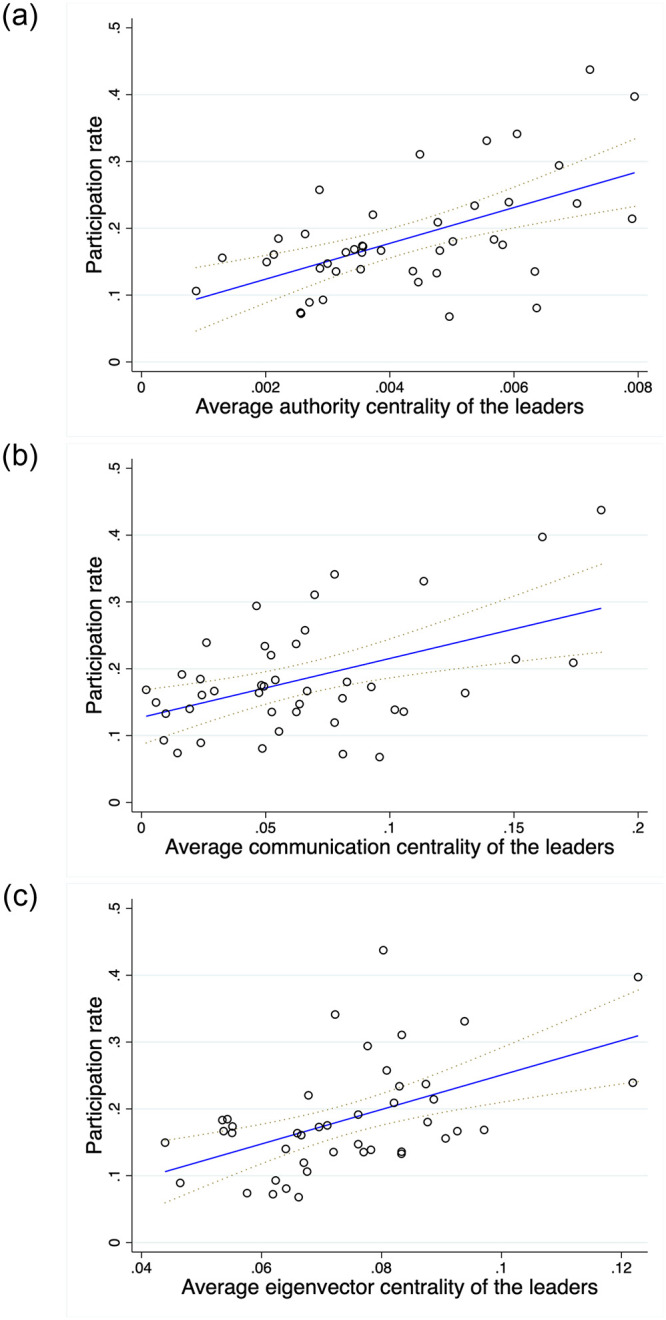
Scatter plots and linear fittings by centrality measures of the leaders. In (a), the vertical axis indicates the participation rate. The horizontal axis shows the average authority centrality of the leaders. The circles represent observations, and the blue line represents a predicted participation rate as a function of the average authority centrality of the leaders. In (b), the vertical axis indicates the participation rate. The horizontal axis shows the average communication centrality of the leaders. The circles represent observations, and the blue line represents a predicted participation rate as a function of the average communication centrality of the leaders. In (c), the vertical axis indicates the participation rate. The horizontal axis shows the average eigenvector centrality of the leaders. The circles represent observations, and the blue line represents a predicted participation rate as a function of the average eigenvector centrality of the leaders.

Following the approach of Banerjee et al. [4], we compare the correlations between the adoption rate and the three centrality measures in Fig 3. Fig 3(a) illustrates the scatter plot of the final participation in microfinance and the authority centrality, and Fig 3(b) and 3(c) presents the scatter plot of the final participation and the other two centralities for comparison. As can be seen, the authority centrality of the leaders is highly correlated with the participation rate, and the correlation (0.562) is higher than the correlation with the other two centralities of the leaders (0.475 for the communication centrality and 0.519 for the eigenvector centrality). With this in mind, in the following session, we present that authority centrality is a more useful predictor than communication centrality with LASSO regression results.

#### LASSO analysis

By conducting LASSO regressions, we confirm here that the authority centrality is more highly correlated with the final participation rate than any other variables, including the communication centrality.

**Fig 4:**
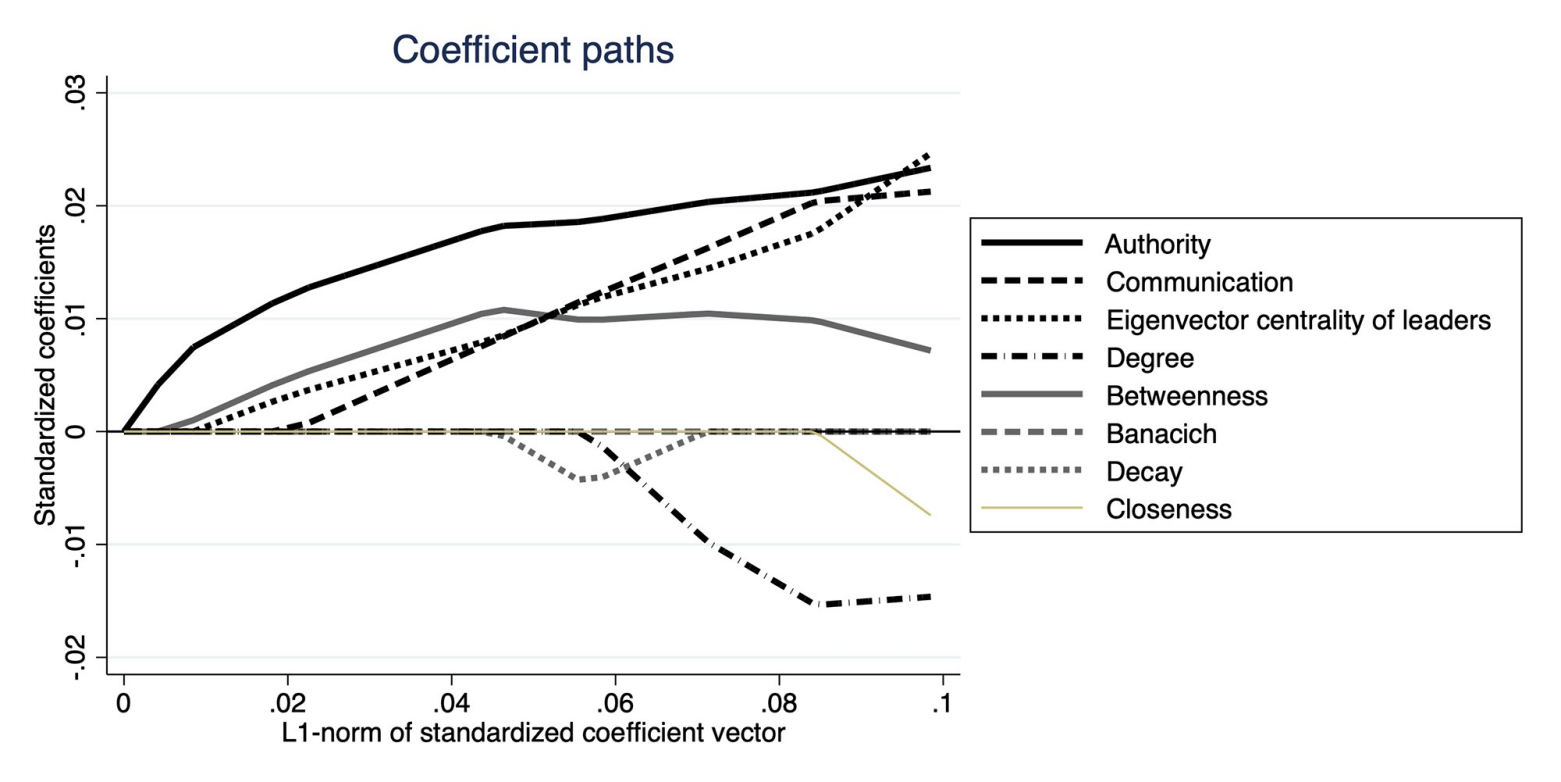
Coefficient paths of coefficients of network centrality variables. In the figure, the vertical axis indicates the standardized coefficients of the network centrality measures. The horizontal axis shows *L*^1^-norm of the standardized coefficient vector. The solid line represents the path of the coefficient of authority centrality of the leaders. Each of the other lines represents the coefficient path of a centrality measure, as shown in the legend of the figure.

Fig 4 plots the coefficient path from a LASSO regression. The model includes variables of the authority centrality, the communication centrality, the eigenvector centrality, and the other five centrality measures considered in the previous regression analyses. In the figure, as the value of the horizontal axis increases, the sum of absolute values of the coefficients can be larger, and so the constraint becomes less restrictive. The coefficients are standardized, and so the unit of the variables does not matter.

**Fig 5:**
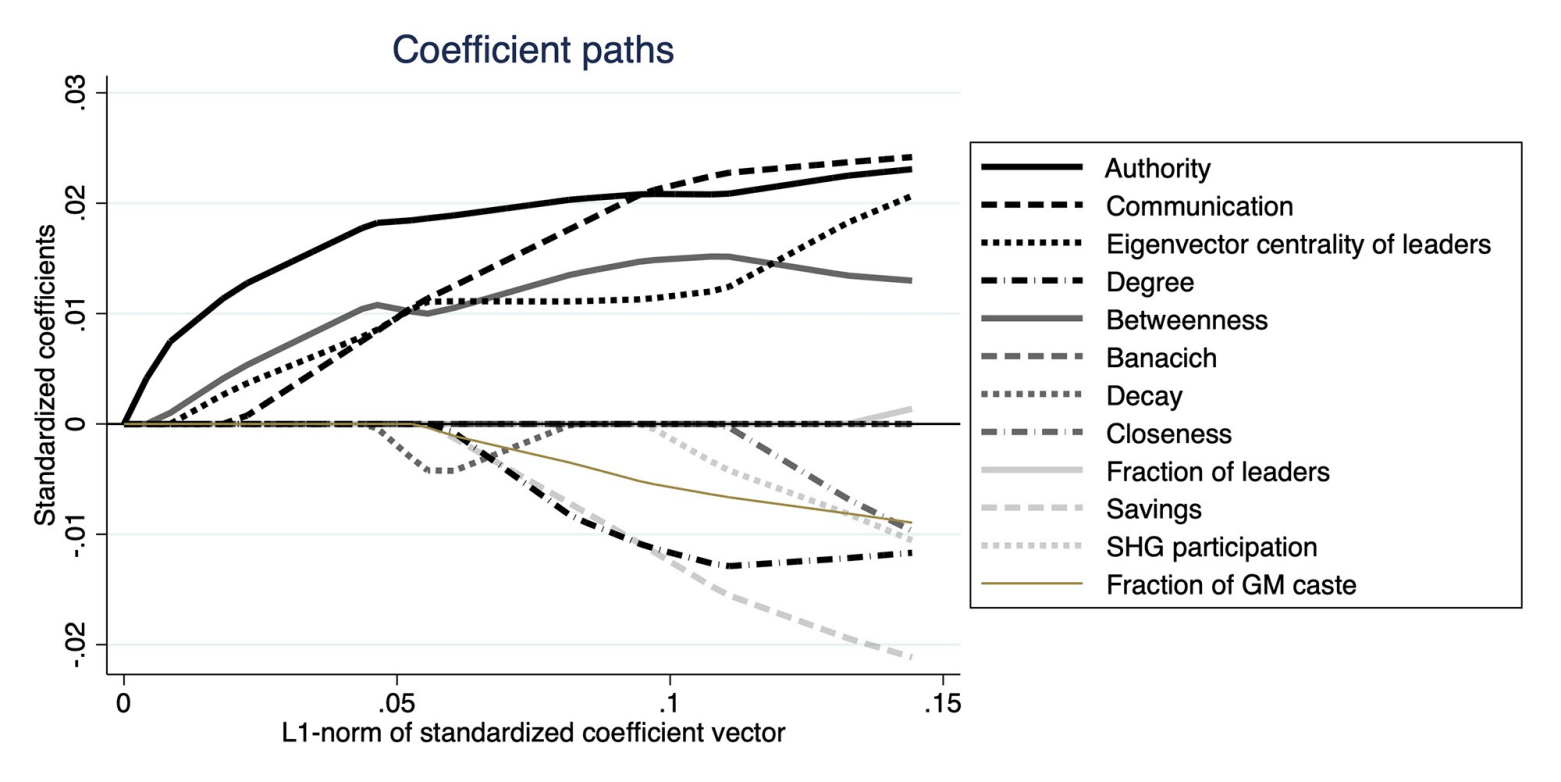
Coefficient paths of coefficients of network centrality and individual characteristics variables. In the figure, the vertical axis indicates the standardized coefficients of the variables. The horizontal axis shows *L*^1^-norm of the standardized coefficient vector. The solid line represents the path of the coefficient of authority centrality of the leaders. Each of the other lines represents the coefficient path of a variable, as shown in the legend of the figure.

Based on the figure presented above, we can observe that the authority, communication, and eigenvector centralities are the primary factors predicting the success of adoption in the data. Among these three factors, the authority centrality holds greater significance as the absolute value of its coefficient is larger than that of the other two measures, except for a few instances of the *L*^1^-norm of the standardized coefficient vector. This result holds even after including additional variables related to leader characteristics, as depicted in Fig 5.

## Discussion

We tested whether hub and authority centralities explain the success of microfinance adoption rates in rural Indian villages. We found that only authority centrality is positively associated with the adoption rates in the data with statistical significance. Moreover, from a series of LASSO regressions, we found that the prediction power of authority centrality is more prominent than other network-based centrality measures.

Our focus on hub and authority centralities is theoretically rooted in Jeong and Shin, in which optimal intervention is characterized by the two centrality measures [6]. In the current paper, however, we found no evidence supporting the idea that providing information to individuals connected with people who spread information well (i.e., agents with high hub centralities) leads to a successful adoption rate. This might result from the fact that our theoretical model assumes a strong homogeneity in the construction of the influence matrix *T*; that is, everyone takes a simple average of their neighbors’ opinions. However, in reality, the weight on one’s own opinion in the learning process could be different across agents depending on their socioeconomic demographics and network characteristics. For this reason, the hub centrality defined in this paper may result in an insignificant factor to explain the adoption rate.

There are other effects that may affect the results in the current paper. For instance, social agents who more frequently interact with others (i.e., high-degree agents) could be more open-minded than others [28]. Consequently, those agents may participate in the microfinance program more actively than others, which may generate an observational learning effect on their neighbors.

It is essential to mention that several crucial points need to be considered. First, the choice of leaders who received the microfinance information was not selected randomly, and this could have led to biased results. In spite of their external selection by Bharatha Swamukti Samsthe, a microfinance institution in rural India, we do not interpret our result as indicating causality between diffusion rates and centrality measurements. Second, the social network data contains inherent measurement errors in various contexts [29–32]. In particular, when network data is sparse, the ordinary least squares estimator might be inconsistent, as recently explored by econometric research [29]. Due to these limitations, the generalizability of our findings may be restricted, and hence, a caveat to our conclusions is warranted.

The network-based target intervention has recently been attracting more attention due to rich social network data [23, 33]. In particular, Beaman et al. provided evidence of one explanation for why network centrality matters in technology adoption behavior via a field experiment [23]. In particular, they argued that many farmers need to learn from multiple people before adopting. Therefore, without a proper targeting strategy at the outset of the diffusion process, the technology adoption rate in a social network may remain perpetually low. In the current paper, we found evidence that authority centrality, which solely requires the information of who interacts with whom, is a useful network-based centrality measure to target to maximize the adoption rate.

## Supporting information

S1 File Replication code. A STATA do file (replication_code.do) contains the replication code. The code produces all the tables and figures in the paper.
(DO) Click here for additional data file.

S1 Data Data file. A STATA data file (replication_data.dta) is required to run the replication code.
(DTA)
